# Length of antibiotic therapy among adults aged ≥65 years hospitalized with uncomplicated community-acquired pneumonia, 2013-2020

**DOI:** 10.1017/ash.2023.248

**Published:** 2023-09-29

**Authors:** Natalie McCarthy, Hannah Wolford, Sophia Kazakova, James Baggs, Brandon Attell, Sarah Kabbani, Melinda Neuhauser, Sarah Yi, Kelly Hatfield, Sujan Reddy, Lauri Hicks

## Abstract

**Background:** The 2014 US National Strategy for Combating Antibiotic-Resistant Bacteria aimed to reduce inappropriate inpatient antibiotic use by 20% for monitored conditions, such as community-acquired pneumonia (CAP), by 2020. Clinical guidelines recommend treating uncomplicated CAP with a minimum of 5 days of antibiotic therapy. Total length of therapy (LOT) >7 days or >3 days after clinical improvement is rarely necessary. In a previous study estimating LOT in uncomplicated CAP patients, 71% of patients ≥65 years exceeded recommended duration of antibiotics in 2012–2013 (Yi et al, 2018). We evaluated annual trends in LOT in adults ≥65 years hospitalized with uncomplicated CAP from 2013 to 2020. **Methods:** We conducted a retrospective cohort study among patients in the CMS database with a primary diagnosis of bacterial or unspecified pneumonia using *International Classification of Diseases 9th* and *10th Revision* codes, length of stay (LOS) of 2–10 days, discharged home with self-care, and not rehospitalized in the 3 days following discharge. Discharge home was used as a surrogate for clinical improvement. Because inpatient LOT is not available in CMS data, we used linear regression to model inpatient LOT as a function of LOS using data on CAP patients ≥65 years from the PINC AI healthcare database. Postdischarge LOT was based on prescriptions filled following discharge. Total LOT was calculated by summing estimated inpatient LOT and actual postdischarge LOT (Fig. 1). Total LOT >7 days and postdischarge LOT >3 days were considered indicators of likely excessive LOT. We reported trends in the proportion of patients with likely excessive LOT during the study period. **Results:** From 2013 through 2020, there were 400,928 uncomplicated CAP hospitalizations among patients aged ≥65 years. Patients were more likely to be female (55%), and they had a median age of 76 years and a median LOS of 3 days. The median total LOT decreased from 9.5 days in 2013 to 7.7 days in 2020. The proportion of patients with total LOT >7 days decreased from 68% in 2013 to 50% in 2020 (% change, −27%); the proportion with postdischarge LOT >3 days decreased from 73% in 2013 to 62% in 2020 (% change, −16%) (Fig. 2). **Conclusions:** Likely excessive total LOT for adults ≥65 years hospitalized with uncomplicated CAP decreased by 27% in 2020, a considerable improvement from 2013. However, the high proportion of patients with likely excessive postdischarge LOT in 2020 (62%) demonstrates the need for antibiotic stewardship to optimize prescribing at hospital discharge.

**Figure:**
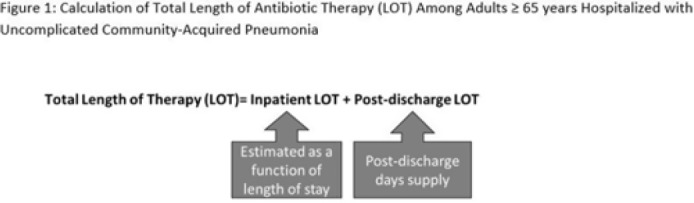

**Figure:**
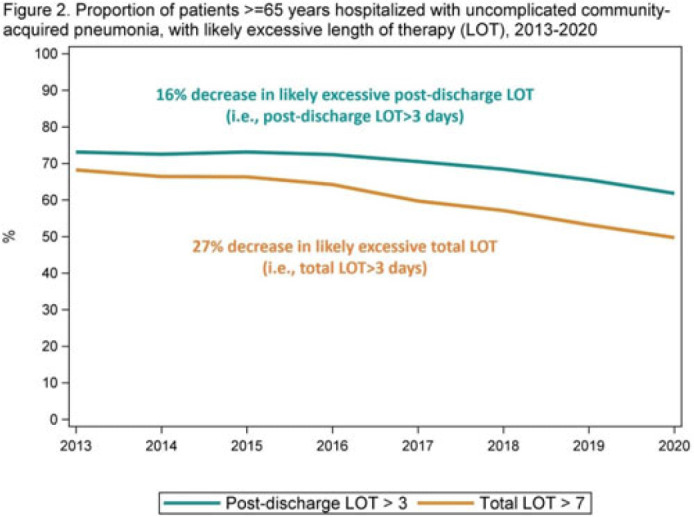

**Disclosures:** None

